# A dynamic nomogram for predict individual risk of malignant brain edema after endovascular thrombectomy in acute ischemic stroke

**DOI:** 10.1038/s41598-024-60083-w

**Published:** 2024-04-25

**Authors:** Huiyuan Wang, Chenghua Xu, Yu Xiao, Panpan Shen, Shunyuan Guo, Yafei Shang, Xinyi Chen, Jie Xu, Chunrong Li, Mingming Tan, Sheng Zhang, Yu Geng

**Affiliations:** 1 Center for Rehabilitation Medicine, Department of Neurology, Zhejiang Provincial People’s Hospital (Affiliated People’s Hospital, Hangzhou Medical College), Hangzhou, 310014 Zhejiang China; 2 School of Clinical Medicine, Graduate School, Bengbu Medical College, Bengbu, China; 3 Department of Neurology, Taizhou First People’s Hospital, Taizhou, Zhejiang China; 4 Department of the Second Clinical Medical College, Zhejiang Chinese Medical University, Hangzhou, China; 5 Department of Quality Management, Zhejiang Provincial People’s Hospital, Hangzhou, 310014 Zhejiang China

**Keywords:** Predictive model, Nomogram, Malignant brain edema, Endovascular thrombectomy, Stroke, Neurology, Cerebrovascular disorders, Stroke

## Abstract

The aim of this study was to develop a dynamic nomogram combining clinical and imaging data to predict malignant brain edema (MBE) after endovascular thrombectomy (EVT) in patients with large vessel occlusion stroke (LVOS). We analyzed the data of LVOS patients receiving EVT at our center from October 2018 to February 2023, and divided a 7:3 ratio into the training cohort and internal validation cohort, and we also prospectively collected patients from another stroke center for external validation. MBE was defined as a midline shift or pineal gland shift > 5 mm, as determined by computed tomography (CT) scans obtained within 7 days after EVT. A nomogram was constructed using logistic regression analysis, and its receiver operating characteristic curve (ROC) and calibration were assessed in three cohorts. A total of 432 patients were enrolled in this study, with 247 in the training cohort, 100 in the internal validation cohort, and 85 in the external validation cohort. MBE occurred in 24% (59) in the training cohort, 16% (16) in the internal validation cohort and 14% (12) in the external validation cohort. After adjusting for various confounding factors, we constructed a nomogram including the clot burden score (CBS), baseline neutrophil count, core infarct volume on CTP before EVT, collateral index, and the number of retrieval attempts. The AUCs of the training cohorts were 0.891 (95% CI 0.840–0.942), the Hosmer–Lemeshow test showed good calibration of the nomogram (P = 0.879). And our nomogram performed well in both internal and external validation data. Our nomogram demonstrates promising potential in identifying patients at elevated risk of MBE following EVT for LVOS.

## Introduction

Stroke is a significant global cause of death and disability. Endovascular thrombectomy (EVT) is currently the best treatment for acute large vessel occlusion stroke (ALVOS), as it can greatly reduce mortality and improve patient outcomes. However, only half of patients who undergo EVT achieve functional independence, and malignant brain edema (MBE) is a severe complication that can occur after the procedure^1^, leading to poor prognosis. Previous studies have confirmed the effectiveness of early decompressive hemicraniectomy^2,3^ in reducing morbidity and mortality in patients with malignant brain edema. Therefore, identifying high-risk patients for MBE can help clinicians make appropriate triage and early intervention decisions, potentially saving patients’ lives.

Predictive factors for post-ischemic stroke brain edema have been widely discussed^4–6^, and reliable early predictive indicators have been identified, such as the Alberta Stroke Program Early CT Score (ASPECTS), age, early consciousness disorders, baseline National Institutes of Health Stroke Scale (NIHSS), atrial fibrillation, hypertension, collateral circulation^7^, baseline blood glucose, and the level of reperfusion after EVT^8,9^.

However, due to individual differences and multiple factors affecting MBE, a single factor cannot effectively predict MBE. Establishing a clinical risk prediction model can effectively identify high-risk populations for MBE at an early stage. Currently, few predictive models for MBE after EVT exist, including nomograms^8–11^ and the ACORNS grading scale^12^. However, these models did not include computed tomography perfusion (CTP) characteristics in their study, which may affect the models’ predictive ability. Previous research has shown that larger core volumes^13,14^, cerebral blood flow (CBF) in the ischemic areas^15^, and hypoperfusion intensity ratio (HIR)^16^ based on CTP can significantly increase the risk of postoperative MBE in EVT patients.

Our study aims to combine imaging data with clinical characteristics to predict potential MBE. Furthermore, this study provides an external validation of the ACORNS scale and compares its predictive performance with our model.

## Methods

### Patient selection

We retrospectively collected data on patients with acute ischemic stroke who underwent EVT at Zhejiang Provincial People’s Hospital between October 2018 and February 2023. The inclusion criteria were: (1) patients aged 18 years or older; (2) Patients undergo head computed tomography (CT) before EVT to exclude hemorrhage; (3) Patients with acute occlusion in the internal carotid artery (ICA) or middle cerebral artery (MCA) within 24 h of onset, treated with EVT, irrespective of the presence of tandem occlusion; (4) patients who underwent CTP scan before treatment; (5) patients who underwent dynamic head CT scans within 24 h of treatment and on 3 or 7 days. The exclusion criteria were: (1) occlusion of the posterior circulation or isolated occlusion of the anterior cerebral artery; (2) severe infection within 7 days before admission; (3) incomplete imaging and clinical data. Importantly, patients who underwent decompression within 7 days were included in the MBE group and were not part of our exclusion criteria.

**Figure 1:**
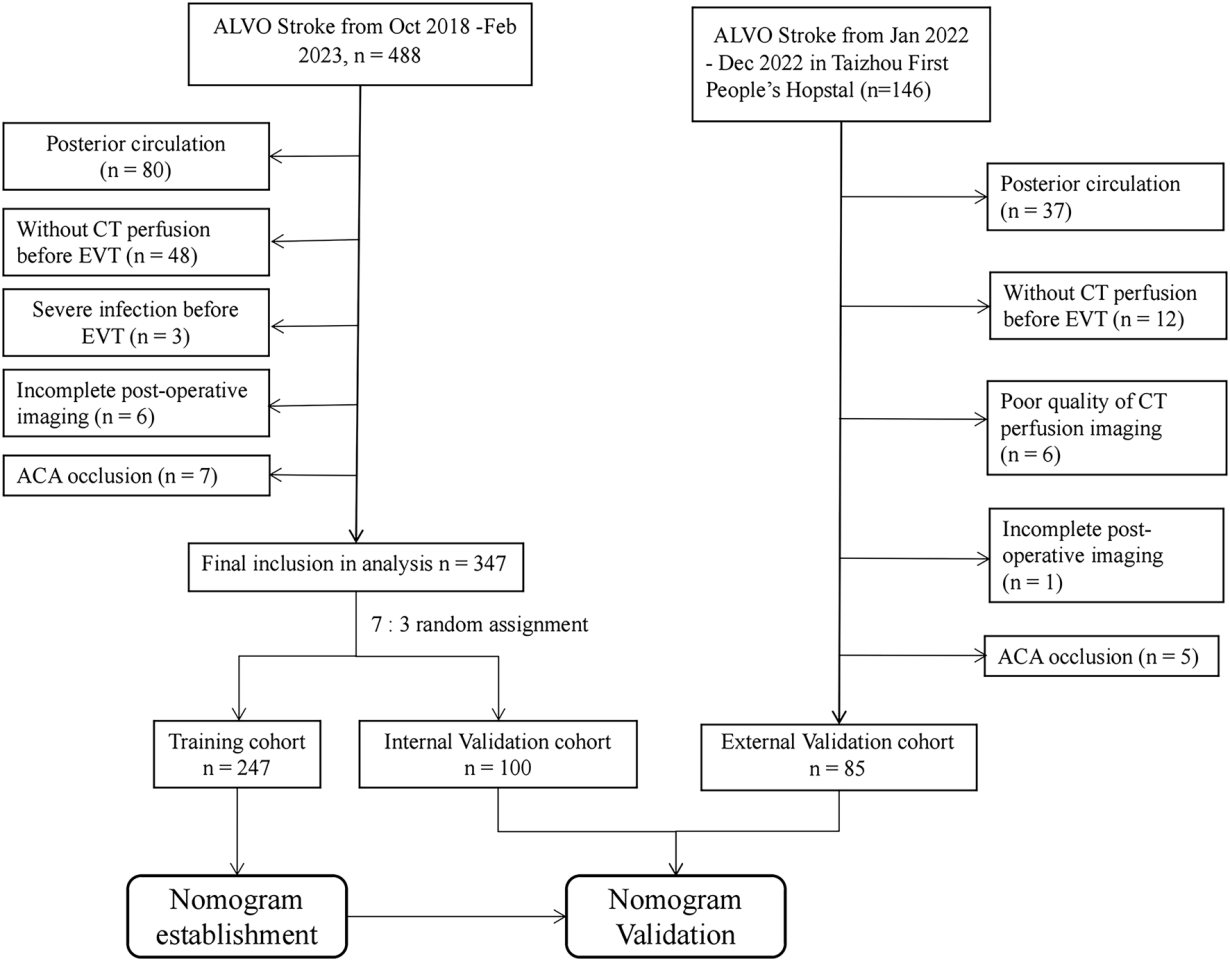
Illustrates the screening process used to identify eligible patients for the study. Flow chart of the study participants’ recruitment. ACA indicated the anterior cerebral artery, EVT indicated Endovascular Thrombectomy.

We also prospectively collected ALVOS patients enrolled with Taizhou First People’s Hospital from January to December 2022 using the same inclusion exclusion criteria. All treatment protocols during the study were performed in accordance with the 2018 Chinese guidelines for ischemic stroke. Figure 1 shows the screening process used to identify eligible patients for the study.

The study was approved by the Ethical Committee of Zhejiang Provincial People’s Hospital (2018KY031) and the Ethical Committee of Taizhou First People’s Hospital (2022KY05002). Prior to treatment, written informed consent was obtained from all patients or their legal representatives for study participation and data utilization.

### Data collection

During hospitalization, we collected baseline characteristics of the patients, including demographic information, medical history (hypertension, diabetes, atrial fibrillation, and preoperative intravenous thrombolysis [IVT]), the site of occlusion, baseline systolic and diastolic blood pressure, and the the Trial of ORG 10,172 in Acute Stroke Treatment (TOAST) classification. We also collected hematological laboratory results, such as baseline blood glucose, white blood cells, neutrophils, hemoglobin, and platelets, along with the admission National Institutes of Health Stroke Scale (NIHSS) score and modified Rankin Score (mRS) at discharge.

All patients undergo EVT under general anesthesia, with femoral artery access chosen as the puncture site. The choice of procedure is determined by the operating physician based on the patient’s condition. If stent retriever thrombectomy is required, the Solitaire AB stent (Medtronic, Irvine, California, USA) is uniformly employed. The EVT operator recorded the time from onset to puncture, time from puncture to reperfusion, the number of retrieval attempts (including the number of direct aspirations and the number of stent retrievers), whether implant stent during the procedure, degree of blood flow reconstruction, and classification of reperfusion level based on the modified Thrombolysis in Cerebral Infarction (mTICI) grading system. Complete reperfusion was defined as mTICI 2b–3.

We recorded imaging features, such as the preoperative Alberta Stroke Program Early CT Score (ASPECTS), clot burden score (CBS)^17^, and collateral score (CS)^18^ on CT angiography (CTA), preoperative CTP core infarct volume and ischemic volume, and collateral index (CI)^19^.

Data collection also encompassed ACORNS score computation for each patient. The ACORNS scale, established by Huang et al. in October 2022, integrates baseline NIHSS, occlusion site, ASPECTS score, collateral circulation status, blood glucose, hypertension history, intravenous thrombolysis, and recanalization grade.

### Imaging evaluation

All patients underwent head CT scans and CTA on admission. Follow-up CT scans were performed at 24 h, 3 days, and 7 days after EVT. All plain CT scans were performed using a single Siemens machine (Erlangen, Germany). Before treatment, ASPECTS scores were calculated. CTA data were acquired using a 320-detector Toshiba scanner (Toshiba Aquilion ONE; Toshiba Medical Imaging, Tokyo, Japan). The CTP data from both stroke centers were processed using the MIStar commercial software (Apollo Medical Imaging Technology, Melbourne, VIC, Australia) with a fully automated processing algorithm that applied singular value decomposition with delay and dispersion correction, generating cerebral blood flow, cerebral blood volume, mean transit time, and delay time (DT). CI ^19^ was defined as the ratio of the volume of DT > 6 s to the volume of DT > 2 s. The occluded vessel was determined based on intraoperative angiography results, and the reperfusion level was classified using the mTICI scale. Two blinded radiologists independently assessed the patients’ imaging data without access to clinical information.

### Definition of MBE

Brain edema was assessed using follow-up imaging CT within 24 h 3 days, and 7 days after EVT. MBE was defined as (1) at least 50% low-density in the middle cerebral artery territory with local brain edema, (2) a midline shift of > 5 mm of the pineal gland or septum pellucidum, or occlusion of the basal cistern.

### Statistical analysis

We randomly selected approximately 70% of patients from our center to form a training cohort, while the remaining patients formed an internal validation cohort, and data collected from Taizhou First People’s Hospital constituted an external validation cohort. We used the training cohort to construct the nomogram and compared the baseline clinical and radiological characteristics of patients with and without MBE in the training cohort. Continuous variables are represented as median (interquartile range), and categorical variables are expressed as percentages. We used the Mann–Whitney U test to analyze continuous variables and the Chi-squared test and Fisher’s exact test to analyze categorical variables. And we assessed collinearity between variables using variance inflation factors (VIFs), with VIF < 10 and tolerance > 0.1 indicating no significant collinearity.

Variables with a P-value less than 0.1 in the univariate analysis were included in the forward stepwise logistic regression analysis, with odds ratios (ORs) and 95% confidence intervals (CIs) calculated, independent predictors of MBE with P < 0.05 were used to construct a nomogram. The area under the receiver operating characteristic (ROC) curve (AUC) was used to assess the ability of the nomogram to distinguish MBE and non-MBE patients in the training and validation cohorts, with the corresponding 95% CI calculated. Calibration plots and the Hosmer-Lemeshow goodness-of-fit test were used to evaluate the consistency between the actual MBE risk and the probability predicted by the nomogram. Additionally, decision curve analysis (DCA) was performed to determine the clinical usefulness and net benefit at different threshold probabilities. We also performed an external validation and compared the predictive performance of the ACORNS scale.

All statistical analyses were performed using IBM SPSS Statistics software (version 26.0; IBM Corporation, Armonk, NY, USA) and R software (version 4.3.1, R Development Core Team, Vienna, Austria). We confirm that we possess a valid copyright license for the SPSS Statistics software and have adhered to all software licensing agreements and regulations. A two-sided P-value of less than 0.05 was considered statistically significant.

### Ethical approval

Ethical approval for this study was obtained from the Ethical Committee of Zhejiang Provincial People’s Hospital (2018KY031) and the Ethical Committee of Taizhou First People’s Hospital (2022KY05002).

### Consent to participate

Informed consent was obtained from all individual participants (or their legal representatives) included in the study.

### Informed consent

Written informed consent was obtained from all subjects before the study.

## Results

We enrolled a total of 432 patients in our study, with 247 patients in the training cohort, 100 in the internal validation cohort, and 85 in the external validation cohort. In the training cohort, the patients had a mean age of 63.0 ± 14.9 years, and 28.3% (n = 63) were female, and the median time from onset to femoral artery puncture was 411 min (279 min–725 min). Among these patients, 39.3% (n = 97) received intravenous thrombolysis (IVT), and 24.0% (n = 59) developed MBE. In the internal validation cohort, 16.0% (n = 16) finally had MBE, and in the external validation cohort, the incidence of MBE was 14% (n = 12). The baseline characteristics of the cohorts are presented in Table 1.

**Table 1:** Baseline characteristics of the population.

Variables	Training cohort	Internal validation cohort	P*
Age, y, mean (SD)	63 (14.9)	67 (13.0)	0.175
Female, n (%)	63 (28.3)	29 (29.0)	0.931
Hypertension, n (%)	162 (65.6)	64 (64.0)	0.849
Atrial fibrillation, n (%)	110 (44.5)	49 (49.0)	0.386
Diabetes, n (%)	50 (20.2)	17 (17.0)	0.524
Stroke characteristics			
Baseline NIHSS score, median (IQR)	16 (12–21)	15 (11–18)	0.034
Thrombolysis, n (%)	97 (39.3)	38 (38)	0.878
ASPECTS, median (IQR)	8 (6–9)	8 (7–9)	0.634
CBS, median (IQR)	6 (3–8)	6 (3–8)	0.795
CS, median (IQR)	1 (1–2)	1 (1–2)	0.134
Baseline SBP, mmHg, median (IQR)	153 (134–172)	149 (137–167)	0.632
Baseline DBP, mmHg, median (IQR)	87 (77–99)	88 (79–99)	0.832
Glucose , mg/dL, median (IQR)	7.21 (6.26–8.55)	6.77 (5.91–8.38)	0.116
Leukocyte, (×10^9^/L), median (IQR)	8.38 (6.63–10.23)	8.6 (6.90–10.91)	0.407
Neutrophils, (×10^9^/L), median (IQR)	6.30 (4.70–8.38)	6.32 (4.90–8.70)	0.799
HB, (×10^9^ /L), median (IQR)	136 (123–150)	135 (124–150)	0.597
PLT, (×10^9^/L), median (IQR)	178 (149–208)	180 (147–225)	0.749
Total ischemic volume, median (IQR)	119 (72–182)	112 (63–172)	0.505
Core volume, median (IQR)	21.5 (8–46.8)	17 (5–57)	0.672
Mismatch ratio, median (IQR)	5.03 (2.82–5.59)	5.25(2.87–10.75)	0.951
Collateral Index, median (IQR)	0.14 (0–0.30)	0.07 (0–0.25)	0.117
Cause, n (%)			
Cardioembolic	116 (45.0)	48 (48.0)	
Large artery atherosclerosis	104 (42.1)	38 (38.0)	
Others	27 (10.9)	14 (14.0)	
Occlusion site, n (%)			
ICA	91 (36.8)	37 (37.0)	0.906
M1–M2	173 (70.0)	69 (69.0)	0.991
Intervention characteristics			
Number of retrieval attempts	2 (1–3)	2 (1–3)	0.750
Implant stent	35 (14.2)	15(15.0)	0.866
OTP, min, median, (IQR)	411 (279–725)	395 (246–642)	0.338
PTR, min, median, (IQR)	57 (40–84)	50 (38–76)	0.184
mTICI 2b-3	240 (97.2)	90 (90.0)	0.008
ACORNS, median, (IQR)	5 (4–7)	5 (3–7)	0.188
MBE, n (%)	59 (24.0)	16 (16.0)	0.157
Decompressive hemicraniectomy	14 (5.7)	8 (8.0)	0.465
Discharge mRS, median, (IQR)	4 (2, 5)	4 (2, 5)	0.374
Total, n	247	100	

*NIHSS* indicates the national institute of health stroke Scale. *SBP* indicates systolic blood pressure, *DBP* indicates diastolic blood pressure. *ASPECTS* the alberta stroke program early CT score. *CBS* the clot burden score. *CS* the collateral score. *HB* Haemoglobin. *PLT* blood platelet. ICA internal carotid artery, Main trunk (M1) and its first-order branch (M2) of the middle cerebral artery. *OTP* the time from onset to puncture. *PTR* time from puncture to recanalization. *ACORNS* indicates the ACORNS grading scale. *SD* Standard Deviation. *IQR* interquartile range. *MBE* malignant brain edema. P*indicates training cohort compared with internal validation cohort.

We investigated the predictors of MBE in the training cohort and observed significant differences between the MBE and non-MBE groups. The MBE group exhibited higher baseline NIHSS, glucose, neutrophil counts, number of retrieval attempts, and white blood cell counts, as well as lower ASPECTS and CBS compared to the non-MBE group. Additionally, CTP parameters such as core volume, ischemic volume, mismatch ratio, and collateral index demonstrated significant correlations with MBE. Furthermore, a higher proportion of patients in the MBE group had a history of atrial fibrillation, ICA occlusion, and implant stent during the procedure (Table 2). We found no collinearity among the variables, except for white blood cell counts (VIF = 11.88). Utilizing forward stepwise regression logic analysis, we identified several independent predictors of MBE, namely preoperative core infarct volume (odds ratio [OR] = 1.049, 95% confidence interval [CI] 1.031–1.067, P < 0.001), baseline CBS (OR = 0.766, 95% CI 0.667–0.879, P < 0.001), collateral index (OR = 0.017, 95% CI 0.001–0.424, P = 0.013), baseline neutrophil count (OR = 1.158, 95% CI 1.032–1.299, P = 0.013), and the number of retrieval attempts (OR = 1.264, 95% CI 1.006–1.588, P = 0.044) (Table 3).

**Table 2:** Univariate analysis of the malignant brain edema in the training cohort.

	Non-MBE	MBE	P value
Age, y, mean (SD)	68 (15.3)	70 (13.3)	0.396
Female, n (%)	52 (31.4)	18 (30.5)	0.836
Hypertension, n (%)	125 (66.5)	38 (64.4)	0.807
Atrial fibrillation, n (%)	75 (40.0)	35 (59.3)	0.008**
Diabetes, n (%)	36 (19.1)	14 (23.7)	0.434
Stroke characteristics			
Baseline NIHSS score, median (IQR)	15 (12–20)	19 (16–24)	0.001**
Thrombolysis, n (%)	77 (41.0)	20 (33.9)	0.333
ASPECTS, median (IQR)	8 (7–9)	6 (5–7)	< 0.001**
CBS, median (IQR)	6 (4–8)	3 (1–6)	< 0.001**
CS, median (IQR)	1 (1–2)	1 (0–1)	< 0.001**
Baseline SBP, mmHg, median (IQR)	153 (134–173)	152 (137–170)	0.754
Baseline DBP, mmHg, median (IQR)	87 (75–100)	85 (74–94)	0.254
Glucose , mg/dL, median (IQR)	7.11 (6.20–8.55)	7.39 (6.62–8.66)	0.333
Leukocyte, (×10^9^/L), median (IQR)	8.03 (6.47–9.77)	9.44 (7.16–11.93)	0.01*
Neutrophils, (×10^9^/L), median (IQR)	5.90 (4.50–7.98)	7.0 (5.60–10.55)	0.002**
HB, (× 10^9^/L), median (IQR)	136 (123–150)	134 (120–150)	0.456
PLT, (×10^9^/L), median (IQR)	182 (150–215)	168 (139–193)	0.042*
Total volume, median (IQR)	105 (66–157)	181 (121–265)	< 0.001**
Core volume, median (IQR)	16 (6–28)	58 (36–104)	< 0.001**
Mismatch ratio, median (IQR)	6.13 (3.74–13.00)	2.49 (1.99–4.00)	< 0.001**
Collateral Index, median (IQR)	0.12 (0–0.28)	0.19 (0.05–0.36)	0.009**
Cause, n (%)			
Cardioembolic	80 (42.6)	33 (55.9)	
Large artery atherosclerosis	87 (46.3)	19 (32.2)	
Others	21 (11.2)	7 (11.9)	
Occlusion site, n (%)			
ICA	58 (30.8)	33 (55.6)	< 0.001**
M1-M2	141 (75.0)	32 (54.2)	0.005**
Intervention characteristics			
Number of retrieval attempts	1 (1–2.75)	2 (1–3)	0.006**
Implant stent	32 (17.0)	3 (5.1)	0.030*
OTP, min, median, (IQR)	430 (270–731)	380 (323–701)	0.882
PTR, min, median, (IQR)	56 (39–82)	62 (43–88)	0.262
mTICI 2b-3	184 (97.8)	56 (95.0)	0.355
ACORNS, median, (IQR)	5 (4–6)	7 (6–8)	< 0.001**
MBE, n (%)	59 (24.0)	16 (16.0)	0.157
Decompressive hemicraniectomy	0	14 (23.7)	
Discharge mRS, median, (IQR)	3 (2, 4)	5 (4, 5)	< 0.001**
Total, n	188	59	

*NIHSS* indicates the National Institute of Health Stroke Scale. *SBP* indicates systolic blood pressure, *DBP* indicates diastolic blood pressure. *ASPECTS* the alberta stroke program early CT score. *CBS* the clot burden score. *CS* the collateral score. *HB* haemoglobin. *PLT* Blood platelet. *ICA* internal carotid artery. Main trunk (M1) and its first-order branch (M2) of the middle cerebral artery. *OTP* the time from onset to puncture. *PTR* time from puncture to recanalization. *ACORNS* indicates the ACORNS grading scale. *SD* standard deviation. *IQR* interquartile range. *MBE* malignant brain edema.

*P < 0.05, **P < 0.01 of the Univariate analysis.

**Table 3:** Multivariate regression analysis for the prediction of malignant brain edema in train cohort.

Variables	Exp (B)	95% CI	P value
Core	1.049	1.031–1.067	< 0.001**
CBS	0.766	0.667–0.879	< 0.001**
CI	0.017	0.001–0.424	0.013**
Neutrophil	1.158	1.032–1.299	0.013*
Number of retrieval attempts	1.264	1.006–1.588	0.044*

Core indicates the Core infarct volumes in CT perfusion. *CI* indicates the Collateral Index, *CBS* the clot burden score.

*P < 0.05, **P < 0.01 of the multivariate regression analysis.

**Figure 2:**
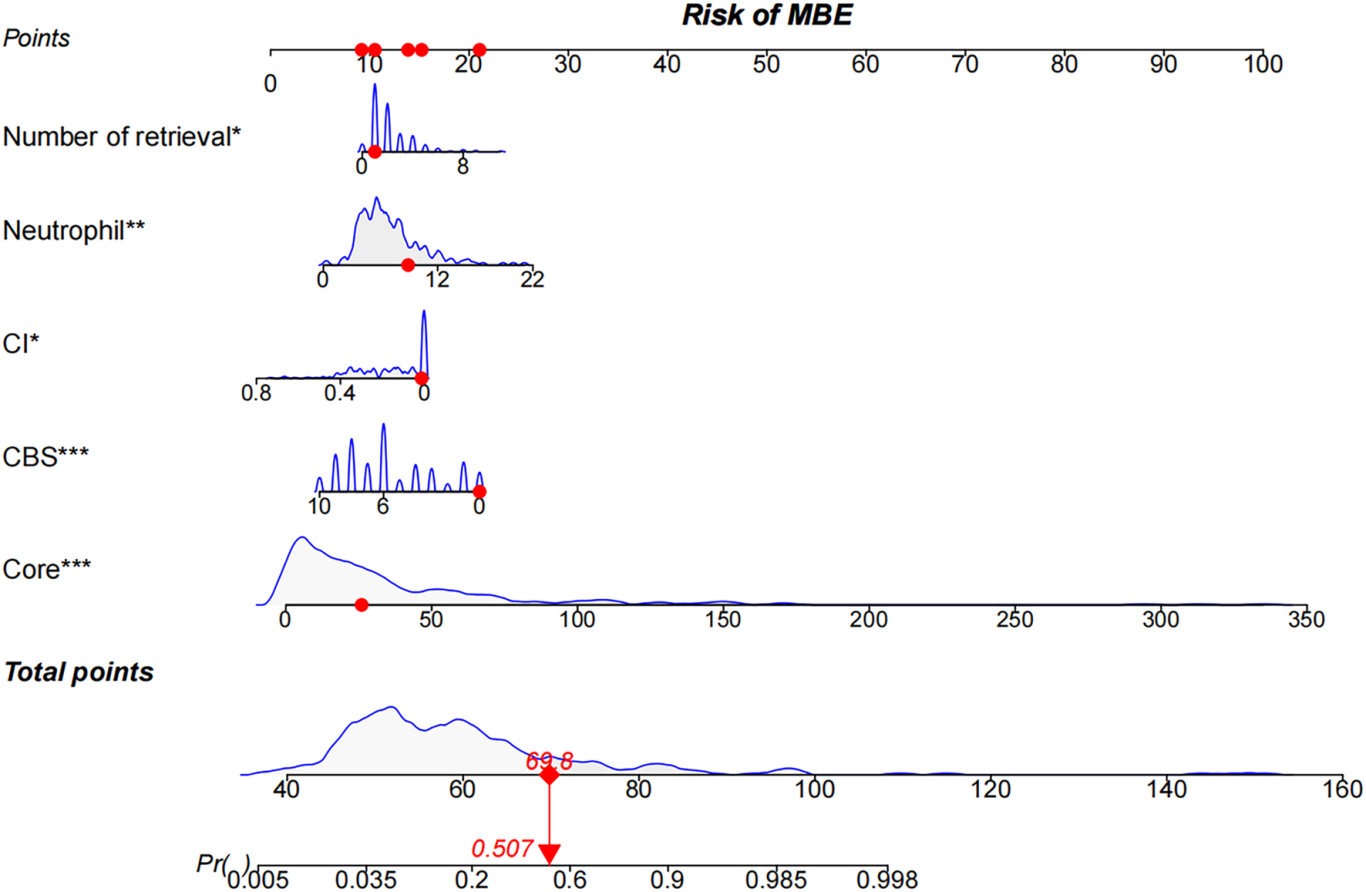
The nomogram model for predicting the probability of malignant brain edema in patients after EVT. The variables, including the number of retrieval attempts, baseline neutrophil, CI, CBS, and CTP core volume could predict the risk of MBE in patients after EVT. EVT, endovascular thrombectomy. MBE, Malignant brain edema. CI, collateral index; CBS, the clot burden Score. Core, CTP core infarct volume. ** indicates P < 0.01, and ***indicates P < 0.001.

**Figure 3:**
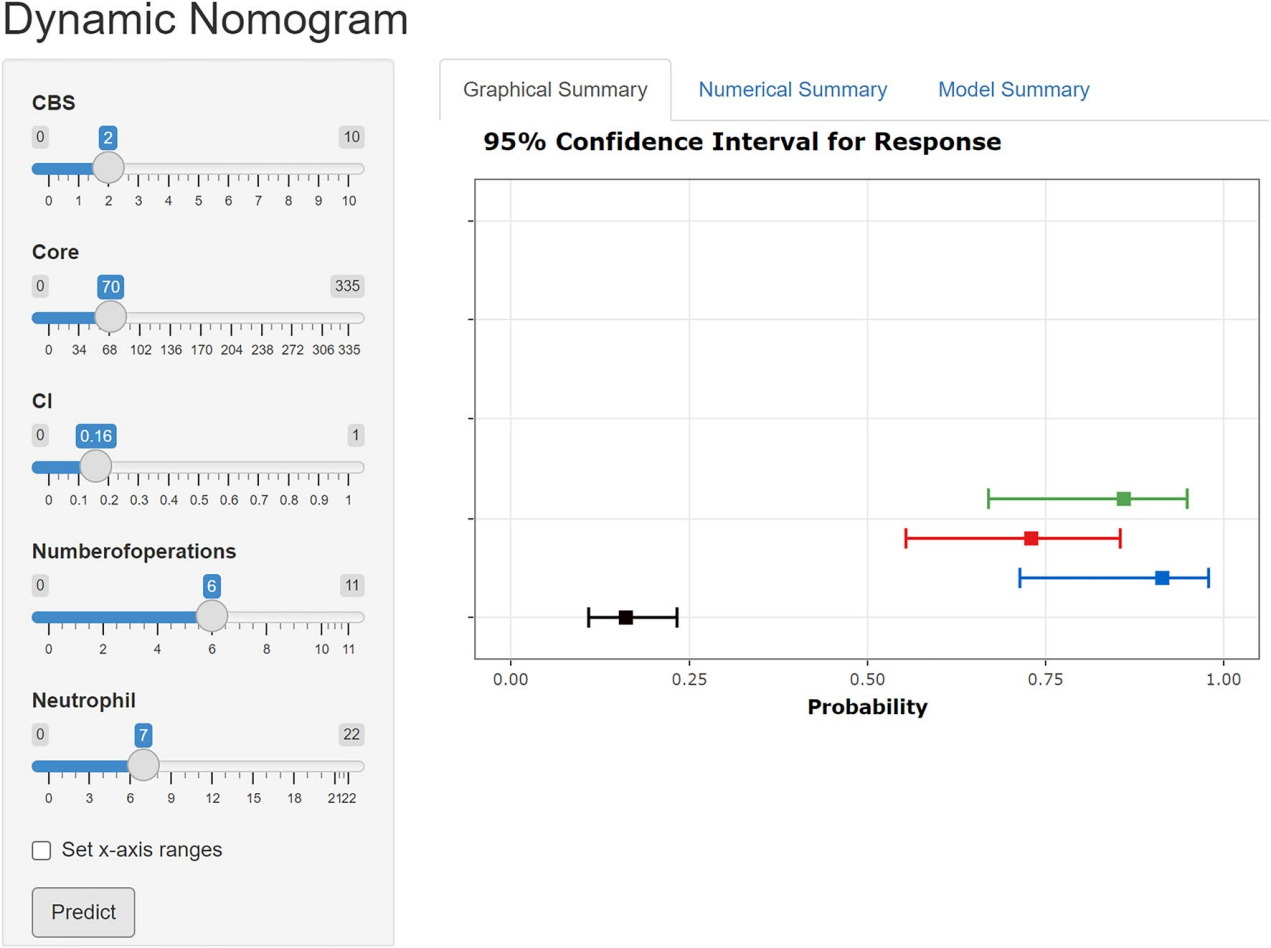
Dynamic nomogram webpage interface for predicting malignant brain edema (MBE). The dynamic nomogram webpage interface, as illustrated in Fig. 3, allows users to adjust parameter options on the left side of the interface. The right side of the interface presents four colored lines, each representing the postoperative MBE probability along with the corresponding 95% confidence intervals for four different patients.

**Figure 4:**
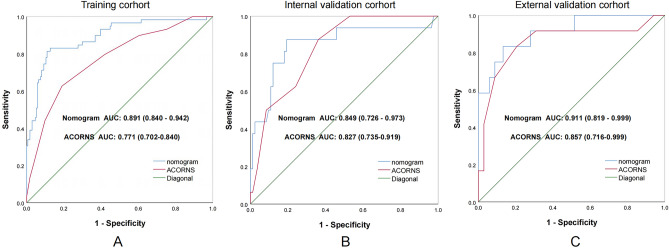
Compare separately in training and validation cohort using receiver operating characteristic (ROC) curves discrimination of our nomogram and ACORNS scale. The model’s ability to differentiate MBE was evaluated by ROC curve, In the training cohort (**A**), the AUC of our nomogram and the ACORNS scale was 0.891 (95% CI 0.840–0.942) and 0.771 (95% CI 0.702–0.840); in the internal validation cohort (**B**), the AUC of our nomogram and the ACORNS scale was 0.849 (95% CI 0.726–0.973) and 0.827 (95% CI 0.735–0.919) and in the external validation cohort (**C**), the AUC of our nomogram and the ACORNS scale was 0.911 (95% CI 0.819–0.999) and 0.827 (95% CI 0.735–0.919).

**Figure 5:**
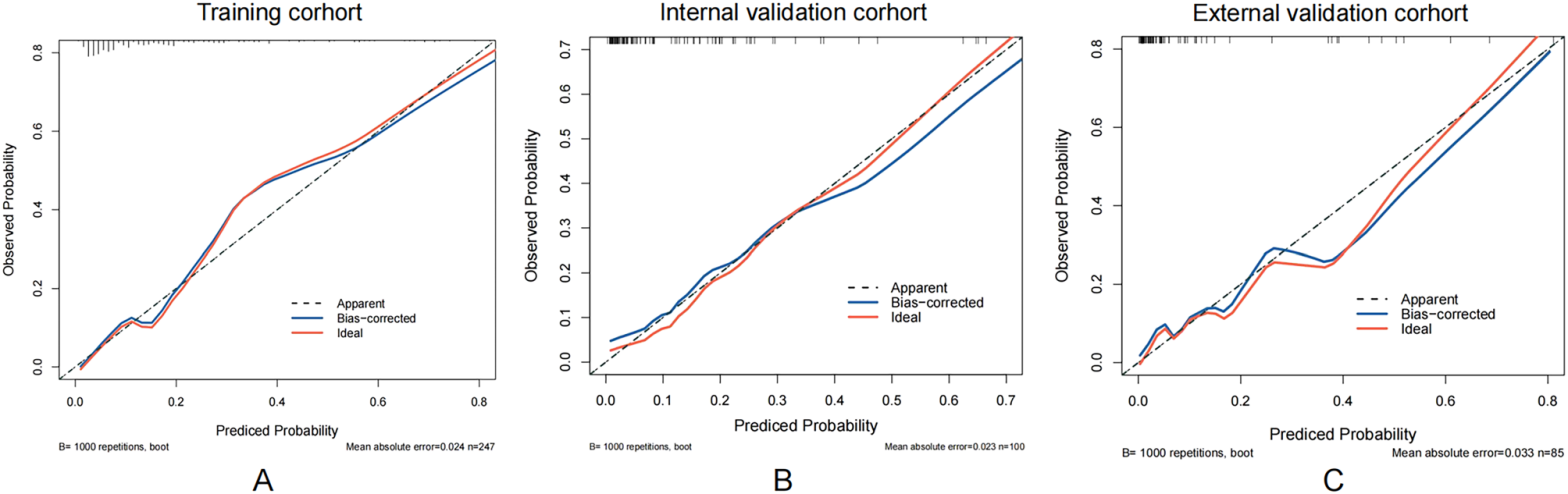
Calibration plot for the nomogram. Ideal is the ideal reference line for the nomogram. Apparent represents the performance of the nomogram, and Bias-corrected represents the performance when bias in the nomogram is corrected.

We developed a nomogram model to predict MBE using four selected features determined by logistic regression analysis (Fig. 2). Figure 3 presents the user-adjustable operational interface of the dynamic nomogram, where the left side allows for item modification. The colored lines on the right represent individual patients’ MBE probabilities along with their corresponding 95% confidence intervals. The interface can be accessed at https://brainedemanomogram.shinyapps.io/DynNom/. The nomogram’s discrimination ability was assessed using the receiver operating characteristic (ROC) curve, with an area under the curve (AUC) of 0.891 (95% CI 0.840–0.942) for the training cohorts, 0.849 (95% CI 0.726–0.973) for the internal validation cohorts, and 0.911 (95% CI 0.819–0.999) for the external validation cohort. In the training cohort, the sensitivity under the maximum Youden index of the ROC curve was 83.1% and the specificity was 84.7%. In the internal validation cohort, the corresponding values were 87.5% and 80.7%, and in the external validation cohort, they were 83.3% and 86.8% (Fig. 4). Hosmer’s test demonstrated a good fit of the model to the data (χ^2^ = 3.755, P = 0.879), and the calibration plot of the nomogram exhibited favorable agreement between predicted and actual MBE risk (Fig. 5).

### Comparison with the ACORNS scale

**Figure 6:**
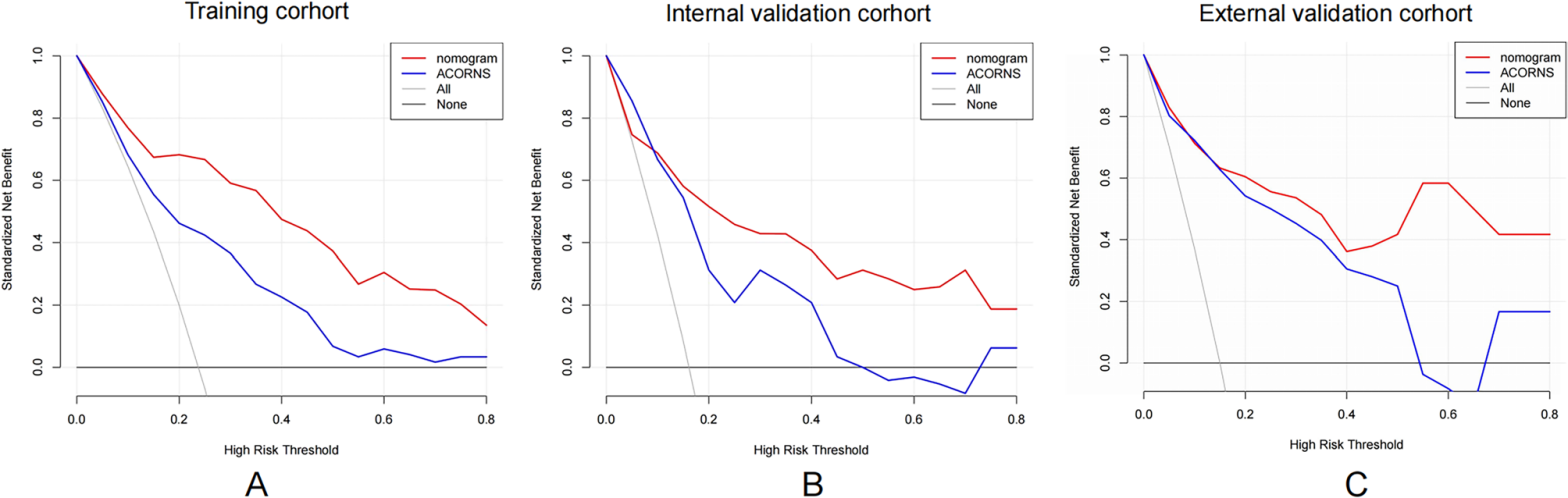
Decision curve analysis (DCA) plots compare the clinical validity of our nomogram and ACORNS scale in the training cohort (**A**) and the validation cohort (**B**,**C**). The DCA plot shows the standard net benefit of both models in predicting malignant brain edema. Across all risk thresholds, our nomogram demonstrates stable clinical net benefits in both training and internal and external validation cohorts, with higher clinical benefits for high MBE risk patients compared to the ACORNS scale.

We conducted an external validation to assess the clinical utility and discriminatory performance of the ACORNS scale developed by Huang et al. , and compared it with our nomogram using DCA and ROC curves. The ACORNS scale is a grading scale for MBE after EVT based on a large sample size. In the training cohort, the ACORNS scale demonstrated an AUC of 0.771 (95% CI 0.702–0.840), with a sensitivity of 64.4% and specificity of 79.9% under the maximum Youden index. In the internal validation cohort, the AUC was 0.827 (95% CI 0.735–0.919), with a sensitivity of 62.5% and specificity of 82.8%. The external validation cohort yielded an AUC of 0.857 (95% CI 0.716–0.999), with a sensitivity of 83.3% and specificity of 75.4% (Fig. 4). Furthermore, we compared the clinical effectiveness of the ACORNS scale and our nomogram using a DCA curve (Fig. 6), which demonstrated that our nomogram consistently generated higher net benefits across all threshold probabilities compared to the ACORNS scale.

## Discussion

In this study, we explored the relationship between preoperative CTP parameters and post-EVT MBE and incorporated these parameters into a nomogram to predict the occurrence of MBE in EVT patients. To facilitate clinical use, we included easily obtainable and evaluatable parameters in our nomogram, which including CBS, preoperative neutrophil count, core infarct volume on preoperative CTP, CI and the number of retrieval attempts. Our dynamic nomogram exhibited good performance in the validation cohort, which proves the reliability of the nomogram and demonstrates better predictive ability and clinical decision-making benefits than the ACORNS scale. Furthermore, our study offers clinicians a user-friendly and convenient dynamic website that enables the prediction nomogram of MBE with high clinical utility.

In our study, the preoperative ASPECT score showed significant correlation in univariate analysis, consistent with previous research^4,12^. However, considering the subjectivity of ASPECTS, we chose to include the more objective and specific core infarct volume in nomogram. Additionly, Our nomogram includes the CBS instead of ICA occlusion. According to the previous studies, the association of ICA occlusion with MBE may be due to ICA occlusion imply a greater thrombus load^20,21^ and poorer collateral circulation. CBS is a simple^17^, objective and easily obtainable parameter that can more specifically reflect the patient’s thrombus load than ICA, can improve the predictive performance of the nomogram.

The status of collateral circulation plays a critical role in the prognosis of AIS patients. Adverse collateral status may diminish the benefit of EVT in LVO patients, increase the risk of intracranial hemorrhage and edema, and impact patient outcomes. Broocks et al^22^. validated the adverse collateral status as an independent predictor of rapid progression malignant edema using the 5-point CS scale based on CTA. Additionally, Murray et al^13^.’s study indicated that higher HIR based on CTP was an independent predictor of early MBE post-EVT. HIR^23^ is the optimal ratio reflecting collateral status calculated using software such as RAPID. For the MIStar software used in this study, Lin et al^19^. also computed the optimal CI ratio reflecting collateral status of brain tissue. Our study incorporates both perfusion collateral status and vascular collateral status into the analysis, and the results show that CI has a stronger correlation with MBE post-EVT compared to CS obtained from single-phase CTA. Therefore, we include CI rather than CS in the nomogram.

Furthermore, the analysis of this study reveals a significant correlation between the number of retrieval attempts during EVT and postoperative MBE, consistent with previous research findings^24^. Prior studies have demonstrated that the proportion of EVT patients achieving favorable functional outcomes decreases with an increase in the number of retrieval attempts during the procedure^25–27^. A procedural frequency exceeding three retrieval attempts may elevate the risk of symptomatic intracranial hemorrhage and unfavorable outcomes postoperatively^28^. Interestingly, this increased risk does not appear to correlate significantly with prolonged procedure duration^26^. The heightened risk is speculated to stem from increased vessel damage resulting from multiple retrieval attempts^29^. However, our study did not provide detailed differentiation of intraoperative procedures, such as direct aspiration versus stent retriever thrombectomy, as these decisions are entirely at the discretion of the operating physician. A more detailed subgroup differentiation could potentially introduce bias into the study.

Our study is in agreement with prior research^30,31^ that reported a higher risk of MBE in EVT patients with an elevated baseline neutrophil count. The underlying mechanism of MBE is linked to systemic inflammation and secondary brain injury^32^. Following a stroke, the blood-brain barrier gets disrupted early on, allowing peripheral immune cells to enter the infarcted tissue, which triggers endothelial dysfunction, local inflammation, and increased brain tissue edema.

The incorporation of core infarct volume of CTP, CBS, CI, and the number of retrieval attempts into the nomogram is a novel feature of our study. Although the effect of reperfusion therapy on edema remains controversial^14,33^, larger core infarct volumes^13,14^ have consistently been associated with an increased risk of MBE in patients with ischemic stroke. Previous models that included ASPECTS have limitations in their predictive ability due to the subjective nature of the score in clinical application. Our study validates the significant correlation between CTP core volume and MBE after EVT and highlights the importance of incorporating this parameter into predictive models for better patient outcomes.

Our dynamic nomogram demonstrated good predictive performance in both the training and validation cohorts. In addition, our study conducted a real-world external validation of the ACORNS scale, which showed that while ACORNS can effectively predict MBE, our nomogram performed better. Additionally, the impact of IVT prior to thrombectomy on post-EVT MBE is still unclear. In our entire study population, IVT was not associated with post-EVT MBE occurrence (P = 0.545), although this result may be limited by our relatively small sample size, further studies are needed to verify whether IVT can promote MBE.

Furthermore, the DCA curve analysis provides a robust approach to evaluating the clinical utility of predictive models. By comparing the net benefits of our nomogram and the ACORNS scale at different threshold probabilities, the DCA curve demonstrated that our nomogram has higher clinical decision-making benefits. Nonetheless, additional validation studies are required to confirm the generalizability and reliability of our nomogram across different populations and settings.

Our study has some limitations. Firstly, our sample size is relatively small, and while we differentiated between training and validation cohorts, the small sample size may affect the reliability of the model. Further external validation is needed to confirm the generalizability and reliability of our model. Secondly, there are numerous clinical and radiological factors influencing MBE, yet our nomogram only incorporates five of these factors. While the inclusion of concise and objective predictive factors is advantageous for clinical applicability, the limited parameters may constrain the predictive performance of our nomogram. Thirdly, our study did not analyze postoperative vascular reocclusion in EVT patients, which could be a significant factor leading to malignant brain edema^34^. Both of our stroke centers did not conduct comprehensive CTA within 24 h postoperatively to assess vascular patency, resulting in the unavailability of this parameter. Future prospective studies could be designed to explore this issue further. Lastly, we excluded patients who did not have CTP data or had poor perfusion quality before surgery, as well as those with incomplete imaging data, which may have introduced some selection bias.

## Conclusions

Our dynamic nomogram, incorporating CBS, neutrophil count, CTP core infarct volume, CTP CI and the number of retrieval attempts, serves as an early predictor of EVT-induced MBE that holds considerable clinical applicability. Additionally, we have developed an operational website for dynamic nomograms, enhancing the accessibility and practicality of the model.

## Data Availability

The data that support the findings of this study are available on request from the corresponding author.
